# Slippage Detection with Piezoresistive Tactile Sensors

**DOI:** 10.3390/s17081844

**Published:** 2017-08-10

**Authors:** Rocco A. Romeo, Calogero M. Oddo, Maria Chiara Carrozza, Eugenio Guglielmelli, Loredana Zollo

**Affiliations:** 1 Research Unit of Biomedical Robotics and Biomicrosystems, Università Campus Bio-Medico di Roma, Rome 00128, Italy; e.guglielmelli@unicampus.it (E.G.); l.zollo@unicampus.it (L.Z.).; 2 The BioRobotics Institute, Scuola Superiore Sant’Anna, Pisa 56127, Italy; calogero.oddo@santannapisa.it (C.M.O.); chiara.carrozza@santannapisa.it (M.C.C.)

**Keywords:** slippage, tactile, sensor, algorithm, prosthetics, robotics

## Abstract

One of the crucial actions to be performed during a grasping task is to avoid slippage. The human hand can rapidly correct applied forces and prevent a grasped object from falling, thanks to its advanced tactile sensing. The same capability is hard to reproduce in artificial systems, such as robotic or prosthetic hands, where sensory motor coordination for force and slippage control is very limited. In this paper, a novel algorithm for slippage detection is presented. Based on fast, easy-to-perform processing, the proposed algorithm generates an ON/OFF signal relating to the presence/absence of slippage. The method can be applied either on the raw output of a force sensor or to its calibrated force signal, and yields comparable results if applied to both normal or tangential components. A biomimetic fingertip that integrates piezoresistive MEMS sensors was employed for evaluating the method performance. Each sensor had four units, thus providing 16 mono-axial signals for the analysis. A mechatronic platform was used to produce relative movement between the finger and the test surfaces (tactile *stimuli*). Three surfaces with submillimetric periods were adopted for the method evaluation, and 10 experimental trials were performed per each surface. Results are illustrated in terms of slippage events detection and of latency between the slippage itself and its onset.

## 1. Introduction

Tactile sensing is fundamental for enhancing performance of robotic systems in unstructured environments. Moreover, in the last years the interaction of robots with the surrounding environment is increasingly moving more and more towards the inclusion of human beings, in addition to inanimate objects [1]. This implies the need of advanced sensing capabilities to effectively interact with people and successfully grasp and manipulate objects. In particular, tactile sensing plays a paramount role to: (a) gather information about the object properties, e.g., shape and hardness, and about the interaction forces, and (b) properly detect slippage phenomena that may occur during manipulation and grasping tasks. Robotic end effectors are the devices at the end of robot kinematic chains. They can be grippers or also robotic hands. They should be able to control slip events in a dexterous manner, and to avoid grasp instability or else damages to the objects. While the robotic end effector performs an action or handles an object, unpredictable events might occur, which hinder the success of the action; the availability of control strategies relying on tactile sensory feedback could greatly increase robot performance when facing such a situation. 

To date, the number of applications using tactile information is limited [1]. Depending on the technology, tactile sensors still suffer from certain drawbacks, such as hysteresis, non-linearity and sensitivity to environment (e.g., temperature). The resulting lack of reliability leads robotic end effectors to mainly act within structured environments, without relying on tactile information. Even when they are endowed with tactile sensory system, the dexterity of the human hand is highly difficult to mimic. Indeed, tactile sensory systems are not capable of measuring all the tactile properties (contact forces, slippage, temperature, roughness, texture, hardness, etc.) as human tactile sensing can do simultaneously. Further, most tactile sensors can only be used either for static or dynamic sensing, but not both. Much research was performed in the last decades, yet the biological tactile system is not satisfactorily reproduced. For this reason, improving tactile information can predispose the robots towards a more natural behavior, including e.g., their reactions to unexpected events and situations. Sensory feedback from tactile sensors would be of great help in many robotic domains, such as robotic manipulation and prosthetics. Nonetheless, the current state of the art regarding commercial prosthetic hands reports only one case of a device provided with feedback, from tactile or force sensors and employed for slip control [2]; this confirms that tactile sensing technologies, including slip detection, are not easily applicable to prosthetic systems yet, despite the considerable efforts of the last years [3]. Moreover, it is worth noticing that the number of research groups and activities focused on tactile sensing has traditionally been lower than on other sensing principles (e.g., artificial vision), thus leading to a slower development of the tactile technology [4]. 

The literature proposes plenty of tactile sensors and procedures for slippage detection. In tactile sensing applications, a critical issue is the integration of static (force/pressure measurement) and dynamic (slip detection) information. The main approach used until the end of last century relied on the calculation of the static friction coefficient from normal and tangential force measurements [5,6]. This requires a multiaxial force sensor in order to measure forces at least along two axes (including the normal one); as an alternative, tangential forces can be analyzed in the frequency domain [7,8] by resorting to Fast Fourier Transform (FFT), Discrete Fourier Transform (DFT) and Power Spectrum Density (PSD), or else estimated by means of Kalman filters [9]. 

Other methods take into account the variations of the center of pressure [10,11]. A tri-axial force sensor featuring a Carbon Micro-Coil (CMC) for slippage detection was fabricated in [12]. A widespread solution is to combine more sensing units for static and dynamic information. To this purpose, force sensors can be jointly used with piezoelectric (such as Polyvinylidene fluoride (PVDF) or lead zirconate titanate (PZT)) sensors for slip management [13,14,15]. Moreover, the employment of accelerometers as slippage sensors was also demonstrated in various scenarios [16,17,18]. 

In [19] a slippage sensor based on conductive rubber was proposed, which was able to detect slippage phenomena through the application of the Continuous Wavelet Transform. However, the aforementioned sensor was not able to measure forces. Further methods exploit the derivative function of the normal component of the estimated force [20], or else, the vibrations in a fluid sealed into the core of a biomimetic sensor [21,22]. This is the case of the commercial sensor BioTac (by SynTouch, Montrose, CA, USA), which is able to provide slippage information by means of a band-pass filtered signal of a fluid pressure measurement in the 10–1040 Hz range.

All the reported works present sensors and/or techniques for the detection of slippage often resorting to force measurement on more axes. In this paper, a new method [23] for the detection of slippage events is proposed; it provides an ON/OFF slip information thanks to online processing of a force sensor output. Simple operations such as digital filtering, rectification and envelope of the input signal are cascaded in order to generate the ON/OFF slip signal. The method can be applied to every type of piezoresistive sensors, regardless the number of force axes: i.e., each available axial component might be processed in the same way, though one is sufficient for the generation of the ON/OFF slip signal. Indeed, the difficulty of measuring slippage events starting only from normal force signal is well known [24]; by applying the proposed method to the voltage output of a simple, low cost mono-axial sensor (i.e., normal axis), it is possible to obtain information about slippage along with the force information itself. An application of the method to mono-axial force sensors for force and slippage control of a prosthetic hand is presented in [25]. 

Here, the proposed method will be explained in detail and its experimental validation on a tactile array including microelectromechanical system (MEMS) piezoresistive force sensors will be illustrated. Each sensor in the array is composed of four sensitive units; hence, 16 “channels” are taken into account for detecting slippage, considered as mono-axial components. The raw voltage from all the channels is employed as input for the algorithm. It is possible to convert such an input into a force signal by using calibration matrices [26]; nonetheless, the voltage signal contains all the necessary information for applying the proposed slippage detection algorithm. This approach is highly convenient for online applications, e.g., a robotic hand control system. Indeed, force computation and slip detection from the voltage output can be performed in parallel rather than subsequently, thus speeding up the process.

During the validation trials, slippage was induced by controlling the sliding velocity of the surface in contact with the sensors, which were embedded into a prosthetic finger (see Section 3). A mechatronic platform operated the surface movement, reproducing in this way the slippage event. The slippage detection method was tested on three surfaces with sub-millimetric periods (ranging from 400 µm to 480 µm), using a set of experiments composed of 10 trials for each surface. Such surfaces were chosen for a first rigorous validation as the one shown in the present paper. However, the method was also tested with flat surfaces having roughness values up to 0.2 µm [23], using different piezoresistive sensors. These tests, along with the experiments carried out with common objects in [25], indicated a scarce influence of the material properties towards the algorithm output.

Even though periodic surfaces were adopted, this paper does not aim at providing a technique for surface pattern discrimination. To this end, literature offers solutions like the one in [27]; nevertheless, slippage identification is not concerned, as greater attention is paid to the roughness encoding by means of MEMS tactile sensors. A biomimetic tactile sensor is used in [28] to understand how human mechanoreceptors code textural information during exploratory tasks. Very limited speeds (0.2 mm/s) were set, thus not investigating slippage conditions. Advanced hardware for composite tactile imaging was developed in [29], allowing to reconstruct spatial profile of applied pressure. Although the considerable technological advance, the study neglected the analysis of slippage phenomena. 

The paper is structured as follows: in Section 2 the slippage detection procedure is presented in detail, describing the different blocks with the main characteristics. Section 3 illustrates the experimental setup that was employed for the evaluation of the method, together with the sensors. In Section 4 experimental data elaboration and results are discussed. Finally, Section 5 points out the conclusions and the future work.

## 2. Slippage Detection Procedure 

### 2.1. Theoretical Description of the Method 

**Figure 1:**
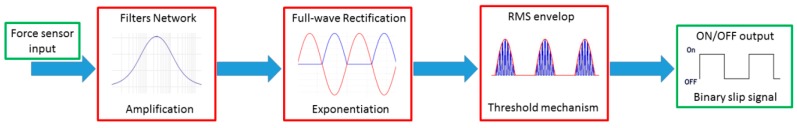
Block scheme of the slippage detection method.

The proposed method consists of three main blocks in cascade between the input signal, which is the raw voltage output of the employed force sensor, and the ON/OFF output, which is the final slippage signal. Figure 1 shows the blocks. Although the calibrated force information is not used as input, it should be noticed that the algorithm output somehow depends from the applied pressure level, i.e., the higher the exerted pressure on the sensor is, the higher the amplitude of the signal available for the ON/OFF signal generation is. However, the applied pressure is directly proportional to the raw voltage, allowing to exploit the latter without loss of information.

**Figure 2:**
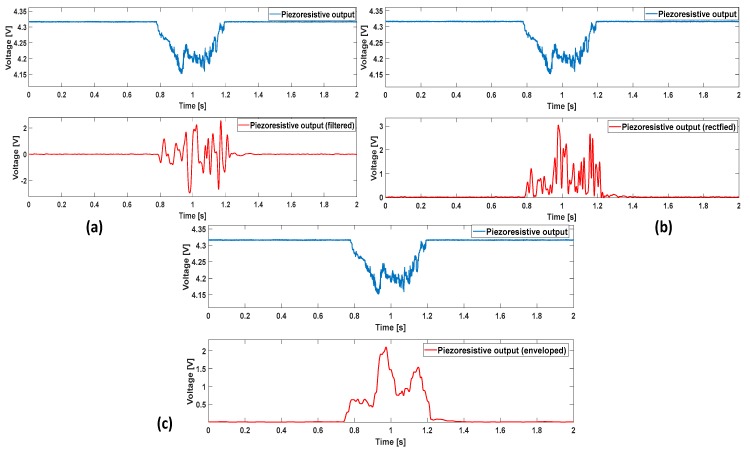
Operations of the algorithm blocks: (**a**) example of voltage signal from a force sensor (**top**) with the corresponding filtered signal (**bottom**); (**b**) same signal as in (**a**) (**top**) with the filtered and rectified signal (**bottom**); (**c**) original voltage signal (**top**) and enveloped one (**bottom**). The signal on top in (**a**–**c**) was obtained by pressing and rubbing the sensor onto an unknown roughness surface. The filters were dimensioned as reported in this section.

The variation of the voltage signal with respect to the baseline (which ranges between 4.3 V and 4.35 V, Figure 2), due to an applied force, is characterized by high amplitude and low frequencies in absence of slippage. When the sensor is slid over a surface (*active touch*), or vice-versa when the surface is slid on the sensor (*passive touch*), the voltage signal carries a higher frequency content and has a smaller amplitude. Such a content can be extracted by means of filtering operations: adequate cut-off frequencies and bandwidth are defined depending on the type of sensor and on its specific structure. Thus, the low frequencies of the original voltage signal are filtered out, by privileging the faster variations produced by slip. At this point, a low amplitude, high frequency signal is generated; the presence of dense fluctuations indicates that the relative movement between the object and the sensor is occurring. The filtering operation is computed together with an opportune amplification (block 1) and is followed by the rectification and exponentiation of the signal (block 2), which is in turn followed by an enveloping phase (block 3). An ON/OFF signal relative to the onset of a slippage event is then generated. The algorithm blocks are described in detail below. 

#### 2.1.1. Filter Network

The filtering operations are responsible for the high frequency extraction from the force (voltage) signals. Thus, it is crucial to accurately design the filter parameters in order to obtain proper performance. A band-pass configuration was chosen for controlling the whole bandwidth, though a high-pass filter can also be adopted. The most general configuration of the method requires one to fix the low cut-off frequency *f_col_* around 7–10 Hz, in order to avoid the signal slow variations due to the applied force. By filtering the signal content up to a high cut-off frequency *f_coh_* equal to 50 Hz, the vibrations generated by the slippage can be adequately isolated, and relayed to the next elaboration stages. Nonetheless, the solution implemented in this work is general; the bandwidth can also be varied, depending on the sensor structure and output.

For the filters network, Infinite Impulse Response (IIR) *causal* filters were designed. IIR filters are computationally more efficient than the Finite Impulse Response (FIR) type, while the causality is fundamental for a real-time implementation. A 4th-order Butterworth filter was employed. It can be expressed by means of its transfer function *H_b_*(*z*) as:

(1) $$H_{b}\left(z\right)=\;G_{b}\frac{\sum_{n=0}^{N}b_{n}z^{-n}}{\sum_{n=0}^{M}a_{n}z^{-n}},$$

and:

(2) $$Y\left(z\right)=H_{b}\left(z\right)\cdot X\left(z\right)$$

is the filtered output in the *z*-domain, with the possibility to control the filtered signal amplitude through a gain *G_b_*. In Equation (1) N = M is the filter order while in Equation (2) *X*(*z*) is the input piezoresistive signal. Equation (1) also gives an idea of how the filter order influences the transfer function. The polynomials complexity augments with the order; this adds delay to the system, even though the filter performance is improved.

Additional digital 4th-order stop-band filters, can be cascaded to the Butterworth one. A flat response in the pass-band region was searched, as well as a quick roll-off transition to the stop-band region. In Figure 2a, a general example of filtered signal is illustrated together with the original unfiltered piezoresistive signal. The choice of high order filters, higher than three, is due to the necessity of achieving fast roll-off. In this way, frequency components outside the range of interest might be easily discarded, including frequencies near the bandwidth limits. Nevertheless, the filter order must be carefully chosen considering Equation (1), avoiding too much elevate values.

#### 2.1.2. Rectification

The filtering stage provides a high frequency signal, yet characterized by a bipolar nature. The rectification can improve the subsequent thresholding. In Figure 2b an example of rectification is provided. By inverting the polarity of the negative spikes, the time window where the signal is ON (i.e., above threshold) can be longer than in the case of a bipolar signal.

A full-wave rectification, or at least a half-wave rectification needs to be performed. Moreover, to privilege the slip signal content with respect to the peaks due to contact and release phases, the exponentiation of the signal amplitude was computed. Indeed, these peaks may sometimes be present although the stop-band filters have a great impact on the signal. The amplitude of such peaks is commonly smaller than the true positives; exponentiation helps to increase the amplitude difference between false positives and true ones. To this end, the filtered and rectified signal can be raised to the third power, and whole process is described by the following mathematical function *H_f+r_*(*z*):

(3) $$H_{f+r}\left(z\right)=\;\sqrt[{3/2}]{H_{f}\left(z\right)\cdot {H_{f}}^{*}\left(z^{*}\right)}$$

where:

(4) $$H_{f}\left(z\right)=\;H_{b}\left(z\right)\cdot H_{e1}\left(z\right)\cdot H_{e2}\left(z\right)$$

i.e., *H_f_*(*z*) represents the transfer function of the overall filters network, comprised of the filters described in Section 2.1.1. The network includes two stop-band filters *H_e_*_1_(*z*) and *H_e_*_2_(*z*), briefly described in Section 3. The filtered and rectified signal *Y*(*z*) becomes:

(5) $$Y_{f+r}\left(z\right)=\;{\left|H_{f}\right|}^{3}\cdot X{\left(z\right)}^{3}=\;\sqrt[{\frac{3}{2}}]{X{\left(z\right)}^{2}\cdot H_{f}\left(z\right)\cdot {H_{f}}^{*}\left(z^{*}\right)}$$

#### 2.1.3. Envelope

Finally, the envelope of the signal is calculated by means of Root Mean Square (RMS). The RMS value *ENV_rms_* is obtained as follows:

(6) $$ENV_{rms}=\sqrt{\frac{1}{W}\sum_{r=w_{1}}^{w_{2}}{x_{r}}^{2}}$$

where *W* = *W*_2_ − *W*_1_ corresponds to the temporal width of the selected window, and *x_r_* is a single sample included within *W*. The rectified signal *Y_r+f_*(*z*) was divided into windows with an equal width *h*; every window *W_i_* almost totally overlaps the adjacent one *W_i_*_+1_, since they are computed as:

(7) $$W_{i}=\left[Y_{r+f}\left(i\right),\;Y_{r+f}\left(i+1\right)\ldots Y_{r+f}\left(i+h\right)\right]$$

(8) $$W_{i+1}=\left[Y_{r+f}\left(i+1\right),\;Y_{r+f}\left(i+2\right)\ldots Y_{r+f}\left(i+h+1\right)\right]$$

i.e., the overlap is shifted by one sample from a window to the next one.

The envelope acts as a low pass filter; it generates a continuous curve starting from the peaks of the rectified signal. Figure 2c gives an example of enveloped signal. At this point, the ON/OFF slip signal can be obtained with a threshold mechanism (see Section 4). More details about the chosen time window will be provided in Section 4.

## 3. Experimental Setup, Protocol and Algorithm Implementation

The described algorithm was applied to the raw voltage outputs of a 2 × 2 array of piezoresistive MEMS sensors that were integrated in a biomimetic fingertip [27]. Each sensor had four piezoresistors implanted at the roots of a cross-shape structure that transduced the forces acting on a mesa. The sensors were covered by a polymeric packaging material (DragonSkin, Smooth-On, Macumgie, PA, USA), with 10 shore A hardness and about 0.8 mm thickness. The area of mechanical contact sensorized by the 16 channels was about 22 mm^2^. The experiments were carried out under a passive touch protocol: the fingertip was static and a plastic plate was put into contact and then slid by means of a 2 degrees of freedom tactile stimulation platform [30]. It allowed implementing trials with versatile reconfiguration of stimulation conditions. Briefly, the platform had horizontal and vertical translational degrees of freedom. The horizontal axis was operated by means of a linear guide (LTP 60.180.0804-02, SKF Multitec, Göteborg, Sweden), actuated with a DC motor (RE35, Maxon Motors, Sachseln, Switzerland), with position tracking error lower than 30 µm up to 25 mm/s sliding velocity. The vertical axis was operated by means of a voice coil (NCC05-18-060-2X, H2W Tech., Santa Clarita, CA, USA), with force tracking error lower than 20 mN [28]. The platform was previously shown not to introduce spurious vibrations that could be perceived by humans. This was confirmed by a set of experiments involving the recording of neural activity of rapidly adapting (Meissner) mechanoreceptors [30]. 

**Figure 3:**
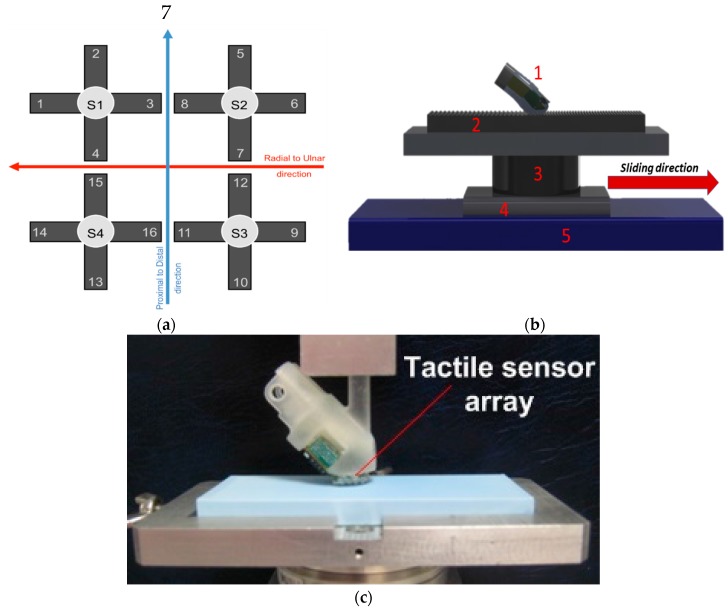
(**a**) Positioning of the 16 tactile units in the fingertip; (**b**) CAD image of the experimental setup. Fingertip embedding the sensing units (1), plastic plate (*stimulus*) (2), voice coil actuator providing interaction force (3) and support (4) moved on a linear guide (5) by a DC motor; (**c**) real picture of the experimental setup.

The purpose of the protocol was to evaluate the effectiveness of the proposed algorithm to: (1) identify the onset of the sliding phase; (2) separate the slippage from the loading and unloading phases that were included in the experimental procedure. During the contact phases the interaction force was kept at 400 mN, and the plastic plate was moved at 6.7 mm/s for 1.5 s and subsequently at 10 mm/s for 1 s. The trial time *T* was set at 6.5 s, as the slippage lasted 2.5 s while 2 s elapsed between the finger contact on the platform and the start of the movement; the same time interval occurred between the end of the slippage and the finger release. Three plates were tested, with 10 experimental repetitions per plate, for the sake of evaluating the generalization abilities of the proposed algorithm with different surfaces. The plates differed for the spatial period Δ*p* (400, 440 and 480 µm) of the pattern of ridges and grooves that alternated along its surface. The height of the ridges of the stimuli was as little as 100 µm to test the capability of the system to identify slippage with a surface having a fine patterning. In this work, all experimental data were acquired at a sample rate of 380 Hz. More details about the experimental setup (Figure 3b,c) can be found in [27]. Additional tests with different piezoresistive sensors and flat surfaces characterized by roughness up to 0.2 µm can be retrieved in [23].

The algorithm was applied to each channel of the biomimetic fingertip, thus generating 16 ON/OFF signals to identify the occurrence of local slippage events. The 16 ON/OFF signals were then grouped taking into account the channels physical orientation; a single global ON/OFF slippage alerting signal was finally generated. Four groups of four channels each were created: (1) *transversal proximal* (TP) orientation; (2) *transversal distal* (TD) orientation; (3) *longitudinal ulnar* (LU) orientation; (4) *longitudinal radial* (LR) orientation. Figure 3a shows the allocation of the sensing units on the biomimetic fingertip, while Table 1 groups them with respect to their orientation. 

For all the four groups, the ON/OFF signal obtained from every channel was put into an AND operator, resulting in four ON/OFF signals. Subsequently, the logical OR operator was applied to the four ON/OFF signals for the determination of the final ON/OFF signal. The basic concept is the following:

1. the presence of local slip phenomena is investigated on every possible direction, both transversal and longitudinal;
2. the AND operator allows reducing the detection of false positives, which mainly occur during the contact and release phases, thus having a robust local slip identification;
3. the OR operator allows detecting the slippage as soon as it is detected on at least one direction, thus converting any identified local slip into a global alert.

The high cut off frequency f_coh_ of the band-pass filter was set to twice the highest expected fundamental frequency (i.e., 25 Hz) based on the experimented spatial periods of the plates Δ*p* and their sliding velocity *v*. However, f_coh_ might be differently chosen without deterioration of the algorithm efficacy, as it will be shown in Section 4. The gain *G_b_* was set to 100 in order to amplify the small vibrations due to slip. Two additional digital 4th-order stop-band filters, i.e., elliptic filters, were cascaded to the Butterworth one. Their transfer functions *H_e1_*(*z*) (*low filter*) and *H_e2_*(*z*) (*high filter*) were set with unitary gain, while the pass-band ripple (peak to peak) and the stop-band attenuation were set to 0.01 dB and 40 dB respectively. They were conceived to inhibit the frequency content below the low limit of the bandpass filter (low filter) and above its high limit (high filter). Rectification (full-wave), exponentiation and enveloping operations were performed as described in Section 2.

**Figure 4:**
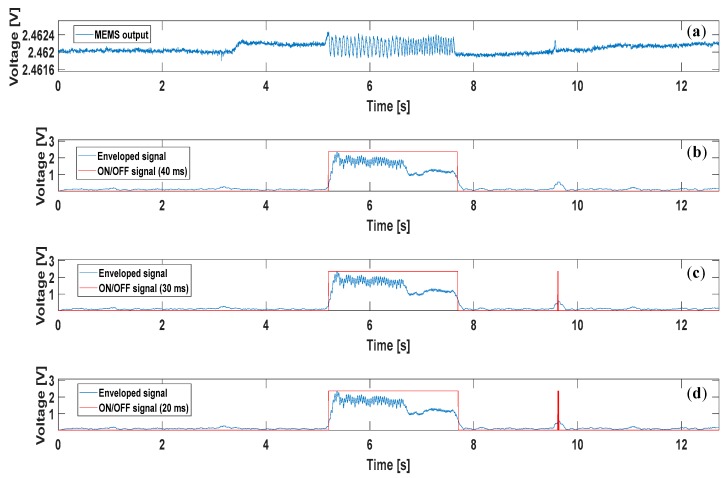
(**a**) Voltage output from one of the 16 tactile units; (**b**) result of the algorithm application using a 40 ms window for the ON/OFF signal computation; (**c**) result of the algorithm application using a 30 ms window for the ON/OFF signal computation; (**d**) result of the algorithm application using a 20 ms window for the ON/OFF signal computation. The enveloped signal was computed using a 40 ms time window in all subfigures. It is clearly noticeable, from the event detected between 9 and 10 s in the last 2 subplots, that the narrowest the window, the highest is the possibility to detect false positives.

As regards the ON/OFF computation, it is important to focus on the width of the temporal window employed. The computational operations described in Section 2, including the final ON/OFF decision, were accomplished by setting the time window width to 40 ms. This value resulted from a trade-off between: (1) the necessity to achieve a computational time below the physiological time needed to respond to slip events (i.e., >70 ms [31,32]); (2) the correct detection of the slippage, reducing the detection of false positives. Figure 4 illustrates the output from one of the tactile units during a trial, and the result of the algorithm (ON/OFF signal) calculated with different time windows. The enveloped signal is considered to be ON if its average value within the time window overcomes an empirically established threshold. The threshold can be adjusted according to the employed sensor technology; it may vary from a kind of sensor to the other. In this case, it was set to 0.5 V.

## 4. Experimental Results

First, it is crucial to evaluate the delay *D* between the beginning of the platform movement with respect to the biomimetic finger, and the OFF to ON transition of the slip signal. The time instant *t_st_* at which the platform starts moving was computed as follows:

(9) $$t_{st}=\left\{t_{1}\in T\;|\;\frac{dx_{pl}\left(t\right)}{dt}\;\neq 0\right\}$$

i.e., the time instant *t*_1_ at which the platform position value *x_pl_* is different from the previous one, is considered as the start of the relative movement during the trial time interval *T*. In other words, the minimum measurable displacement (according to the sampling frequency) has been regarded as the commencement of the slippage. In this way, a rigorous evaluation was carried out, with movements lower than 5 µm considered as the start of the slippage phase. 

As already explained in Section 3, the time window for the algorithm execution was regulated accounting for the physiological response time as well. Similarly, the delay *D* has to be lower than 70 ms, possibly not greater than 50 ms as *D* does not include the efferent response (i.e., the reaction to the identified slip), but only the afferent one. This means that slippage detection has to be followed by an opportune reaction (either of the robot or the human) in terms of force adjustment. Table 2 summarizes the mean values *mD_i_* for all the trials carried out for the three Δ*ps*. These values were obtained based on the position measured by an optical encoder embedded in the mechatronic platform. They were computed with respect to *t_st_* as described by Equation (9).

All the values in Table 2 were calculated as:

(10) $$mD_{i,\Delta \mathrm{pj}}=\;\frac{\sum_{k=1}^{16}ch_{k}}{16}$$

where *i* is the trial number, Δ*p_j_* is the corresponding Δp and *k* is the channel number. Such values resulted to be acceptable (<50 ms) in most cases. These data can be further synthetized by observing the mean values *mD*Δ*p* for every Δ*p*, i.e., the sum of the *mD_i_*_,Δpj_ in every column of Table 2 divided by the number of trials performed per every Δ*p* (i.e., 10). They are:

- mD400 = 36.02 ± 3.91 ms;
- mD440 = 41.78 ± 10.78 ms;
- mD480 = 48.90 ± 9.91 ms.

These results confirm the capability of the proposed slip detection algorithm, in terms of promptness. The *mD*Δ*p* value seems to increase proportionally to the stimulus spacing Δ*p*, coherently with the fact that the output of the sensors shifts towards lower frequencies (thus requiring higher latencies for being observed).

**Figure 5:**
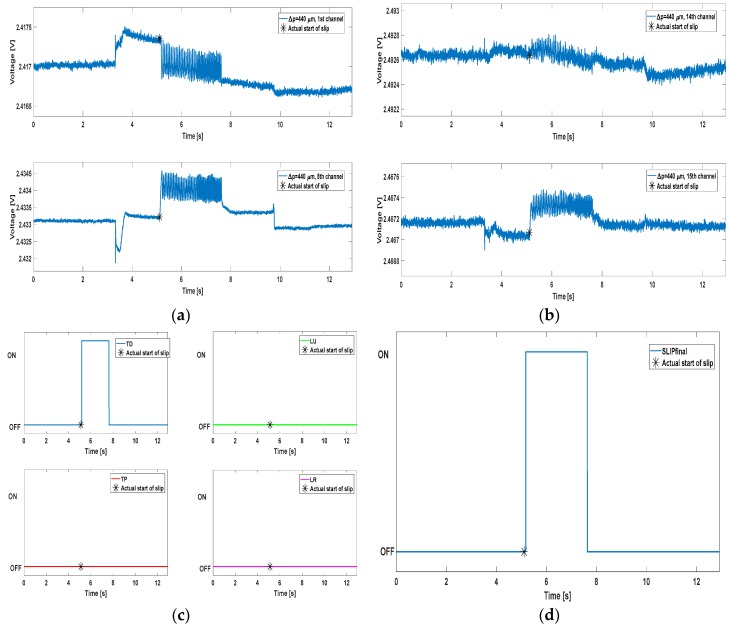
Algorithm performance plots. Panels (**a**,**b**) show the MEMS sensors output from some of the channels, one for each of the four groups mentioned in Section 3; panel (**c**) contains the AND mechanism results for every group; panel (**d**) represents the final slip signal (OR of all the ANDs).

Another crucial feature that should characterize an algorithm for the prevention of slippage events regards the correct avoidance of false positives. While grasping an object, false positives might principally occur during the contact and/or release phases. In Figure 5 some examples of the algorithm performance are shown, extracted from one of the trials relating to the 440 µm stimulus. A channel from each of the four groups mentioned previously is displayed in Figure 5a,b; Figure 5c shows the result of the AND mechanism for all the four groups, whereas Figure 5d depicts the final ON/OFF slip signal generated by means of the OR operation applied onto the four ANDs. In every plot, a black asterisk indicates the start of the mechatronic platform movement *t_st_* (start of slip), calculated according to Equation (9).

**Figure 6:**
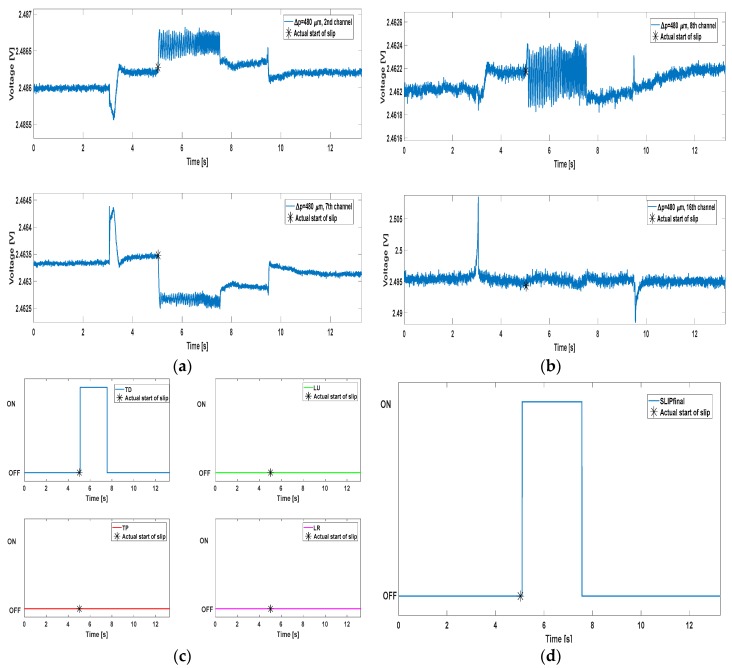
Algorithm performance plots. Again, Panels (**a**,**b**) show the MEMS sensors output from some channels (one per group) while Panel (**c**,**d**) depict respectively the AND mechanism results for every group and their OR, i.e., the final slip signal.

Figure 5c shows how three of the four ANDs result in an OFF signal for the entire duration of the trial. This condition highlights the capability of the algorithm to detect the occurrence of local slip events, without requiring the activation of the whole set of channels. This resembles the biological condition, in which the human finger tactile areas might be involved differently even when performing the same task. The robustness of the algorithm always allowed detecting the true positive, as it is sufficient to achieve the ON at least on one of the four ANDs. In fact, Figure 5d illustrates the ON signal, which covers the whole duration of the slip event. No false positives were detected in the example shown in Figure 5. The same considerations can be extended to Figure 6, which relates to a trial carried out onto the 480 µm stimulus.

In Figure 6, the MEMS signals were taken from different channels with respect to Figure 5. Different shape can be noticed as well: this is due to the various contact conditions that every tactile array unit faces, as already referred. Most notably, channel 16 is characterized by huge peaks in correspondence of the contact and release instants, with no useful content during the slip event. 

The overall success rate of the algorithm in identifying slippage events was equal to 100%, as it correctly recognized the slippage event in all 30 trials. False positives were rarely detected by the algorithm; for evaluating their detection, the time lapse *t_fp_* when false positives can occur has been considered, which is the trial time *T* (i.e., 6.5 s) decreased by the actual slippage event duration (i.e., 2.5 s). The mean percentage of false positives, computed with respect to *t_fp_*, resulted to be: 1.01% (Δ*p* = 400 µm), 0.11% (Δ*p* = 440 µm), and 0.88% (Δ*p* = 480 µm).

**Figure 7:**
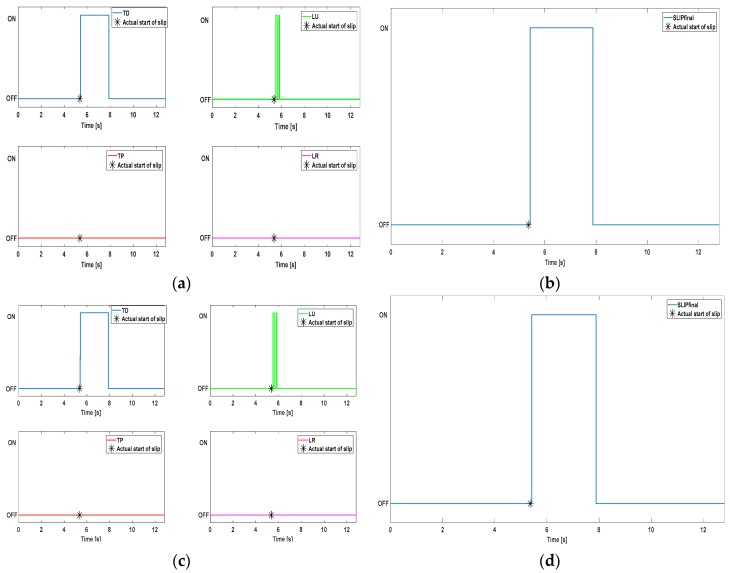
Algorithm performance for different design choices. Panels (**a**,**c**) plot respectively the AND results of the four channel groups. Panels (**b**,**d**) plot the final slip signal generated through the OR operator.

In order to assess relevance of the proposed method, additional tests were performed with different configurations of the filter network. The bandwidth of the bandpass filter has been extended up to 150 Hz, close to the Nyquist limit (i.e., 190 Hz). The reduction of the filters quantity has been tried as well. In this way, rising the slippage speed does not compromise the functioning of the algorithm. Moreover, the possibility to modify the design choices described in Section 2 and Section 3 can be demonstrated. In Figure 7, the algorithm output is shown for a further trial (440 µm). Results depicted in Figure 7a,b were obtained with a bandwidth [10–100] Hz for the bandpass filter, while Figure 7c,d offer the same graphs but relating to a bandwidth [10–150] Hz. In both configurations, the stopband filter *H_e2_*(*z*) was removed from the filter network.

It is easy to observe that data illustrated in Figure 7 do not significantly differ from the ones of Figure 5 and Figure 6. Causal filters introduce an amount of delay in the system: therefore, removing one of them has the advantage of reducing the delay *D* between the slippage onset (*t_st_*) and the ON signal. E.g., a *D* of 15.31 ms was achieved for the trial of Figure 7. Nevertheless, the absence of a stopband filter unavoidably results into a greater probability to detect false positives. Finally, the effectiveness of the algorithm at higher slippage velocities than the ones used in this work was proved in [23] (up to 8 cm/s) and [25] (up to 4 cm/s).

## 5. Conclusions and Future Work

In this paper, a novel method for detection of slippage events was proposed. Said method was presented through a detailed description of its sub-blocks, which include simple operations such as digital filtering, rectification and envelope of the input signal. Such sub-blocks are cascaded to generate the ON/OFF slippage identification signals. The method was accurately validated on piezoresistive MEMS sensors; an ad hoc automated setup was experimented for this purpose. A total of four sensors, integrated into a biomimetic fingertip, were employed; each sensor featured four channels, leading thus to sixteen channels for the slippage detection analysis.

Relative movements lower than 5 μm between the sensor and the contact surface were detectable through the developed algorithm. True positives were detected in 100% of the cases and minimum average delays resulted lower than 50 ms. False positives have been detected in no more than 1.01% of the cases. Remarkably, it is not necessary to employ the force value to apply the method: the raw voltages of sensor outputs (relating to the force) can be used as input. In this manner the force estimation and the ON/OFF slip signal generation can be performed as parallel operations rather than consecutive.

Future works will focus on the use of additional sensors for the algorithm validation, with the aim of extending the algorithm validity beyond the piezoresistive domain. Surfaces with more naturalistic texture, in place of regular gratings, will be employed to hopefully confirm the possibility to apply the algorithm regardless the surface characteristics. The method was already tested in a prosthetic control with common objects [25] and with surfaces whose roughness was a priori known [23]; yet further verifications are needed in order to show the general extendibility of the algorithm. 

## Figures and Tables

**Table 1:** The four channel groups.

Channels	Name	Acronym
14, 16, 11, 9	Transversal Proximal	TP
1, 3, 8, 6	Transversal Distal	TD
5, 7, 12, 10	Longitudinal Radial	LR
2, 4, 15, 13	Longitudinal Ulnar	LU

**Table 2:** This table contains the mean delays *mD_i_* of every trial, obtained upon all the 16 channels for every Δ*p*.

Trial	*mD* (Δ*p* = 400 µm) (ms)	*mD* (Δ*p* = 440 µm) (ms)	*mD* (Δ*p* = 480 µm) (ms)
1	43.37 ± 32.98	66.66 ± 15.89	73.48 ± 30.95
2	33.19 ± 22.01	55.44 ± 13.06	44.01 ± 11.73
3	33.19 ± 22.99	42.57 ± 15.98	55.98 ± 12.40
4	39.60 ± 22.01	37.29 ± 13.16	40.92 ± 10.79
5	34.32 ± 20.66	36.30 ± 14.32	47.96 ± 8.74
6	33.19 ± 19.54	39.27 ± 15.76	50.16 ± 6.99
7	35.83 ± 23.10	34.98 ± 11.44	43.56 ± 10.57
8	37.33 ± 25.11	33.99 ± 13.55	39.16 ± 17.35
9	30.55 ± 11.74	36.30 ± 12.71	44.88 ± 16.59
10	39.60 ± 25.17	34.98 ± 14.91	48.84 ± 16.34
